# Mimicking Sub-Structures Self-Organization in Microtubules

**DOI:** 10.3390/biomimetics4040071

**Published:** 2019-10-18

**Authors:** Sanjay Sarma O. V., Sruthi Palaparthi, Ramana Pidaparti

**Affiliations:** 1 College of Engineering, University of Georgia, Athens, GA 30602, USA; sanjaysarmaov@uga.edu; 2 Department of Computer Science, University of Georgia, Athens, GA 30602, USA; sruthi.p@uga.edu

**Keywords:** microtubules, microtubule associated proteins, self-organization, swarm engineering, swarm intelligence, game engine, protofilaments

## Abstract

Microtubules (MTs) are highly dynamic polymers distributed in the cytoplasm of a biological cell. Alpha and beta globular proteins constituting the heterodimer building blocks combine to form these tubules through polymerization, controlled by the concentration of Guanosine-triphosphate (GTPs) and other Microtubule Associated Proteins (MAPs). MTs play a crucial role in many intracellular processes, predominantly in mitosis, organelle transport and cell locomotion. Current research in this area is focused on understanding the exclusive behaviors of self-organization and their association with different MAPs through organized laboratory experiments. However, the intriguing intelligence behind these tiny machines resulting in complex self-organizing structures is mostly unexplored. In this study, we propose a novel swarm engineering framework in modeling rules for these systems, by combining the principles of design with swarm intelligence. The proposed framework was simulated on a game engine and these simulations demonstrated self-organization of rings and protofilaments in MTs. Analytics from these simulations assisted in understanding the influence of GTPs on protofilament formation. Also, results showed that the population density of GTPs rather than their bonding probabilities played a crucial role in polymerization in forming microtubule substructures.

## 1. Introduction

Microtubules (MTs), are highly dynamic polymers undergoing continuous assembly and disassembly in a biological cell. They resemble hollow cylinders with the heterodimers arranged on their walls in chains, growing up to a length of 50 µm [1]. Heterodimers are made of globular proteins called Tubulins. Each dimer is identified to have one α–tubulin and one β–tubulin and these elemental structures alternate to form strings also called protofilaments, rings and other substructures leading to the formation of an MT [2]. MTs play a crucial role in intracellular functions like separation of chromosomes during mitosis, organelle transport, cell locomotion through cilia and flagella [3], by associating themselves with various Microtubule Associated Proteins (MAPs).

Many hypotheses regarding self-organization and functionality of MTs have been proposed over the past two decades and MTs continue to be a major focus of research in molecular cell biology and molecular biology [4]. Though previous research has focused heavily on understanding these complex processes, not much research has been done in understanding the intelligence behind the complex intracellular organization in MTs. Understanding this intelligence through simulations can support researchers in exploring many unknown processes like the effect of various cancer drugs at a cellular level [5]. However, the primary challenge is in designing a framework that can emulate the real-world behavior of MTs and demonstrate the framework’s effectiveness through dynamic models.

Many of the self-organization models and the interactions of MAPs and GTPs proposed so far were developed through experimental observations and complex molecular dynamics simulations. In some cases, practical observations were not possible because of the limitations in microscopy [6,7,8,9,10]. Moreover, molecular dynamics simulations require heavy computations and involve a high level of complexity for simulating even simple behaviors [11,12,13]. In contrast to the molecular dynamics based simulations, we propose an agent-based model, mimicking the proteins as agents by maintaining a certain level of abstraction in their behavior. In our current work, a behavior-based design method as formulated by Brambilla et al. [14] was followed in designing rules for our agents behaving as tubulins and heterodimers. These swarm agents are expected to interact spatially and connect through morphogenesis [6].

The current study is among a few in the literature to use a swarm engineering approach to simulating MT self-assembly process in forming 2D sub-structures. The framework draws inspiration from the work of Gutmann et al. [15] in simulating gliding behavior in MTs in a real-time 3D environment, where gliding is driven by motor proteins and forms dynamic rings and bundle structures. However, it involves millions of computations, posing a challenge to real-time simulations. This obstacle was overcome by using general-purpose computing on graphics processing units (GPGPU) in their work. Rather than developing graphic and simulation computations from scratch, in our study we utilized an off the shelf game design platform Unity [16] in order to reduce the physics computations and overcome the rendering challenges.

Other works along these lines include Nicolas et al.’s [17] numerical simulation of self-organization of MTs, reproducing the molecular diffusion in a chemical process by varying concentrations of tubules on growing and shrinking ends inducing directionality in the system. Their work also simulated the effect of gravity on MT self-organization.

Jun et al. [18] focused on the self-organization of cortical MTs, in which MTs in the 2D cortex of plant cells were simulated demonstrating the effect of inter MT collisions on de-polymerization and their self-organization into parallel arrays due to their polarity. Also, the effect of several mutants destroying the self-organization was analyzed in their work. The effect of spatial dimensionality on MT self-organization was investigated by Jamie et al. [19]. They conducted simulations on filament active networks and motor-proteins self-organizing into tubes in 2D and 3D spaces. The results from their research clearly demonstrate how simple simulations with user-modifiable parameters can help visualize various outcomes and also how the simulation outcomes are affected by the modeling choices.

The objective of our current study is to develop a simulation framework mimicking the swarm engineering concepts and investigate the self-organization of 2D sub-structures of MT. First, we modeled the general behavior of MTs in self-organizing tubulins, forming lower level structures like protofilaments and rings. We analyzed these models assimilating the interaction of GTPs and GDPs with tubulins as proposed in the literature [1,6,7]. Results obtained from the simulations of sub-structure formation are presented and analyzed for GTP concentration, GTP bonding probability and the effect of nucleotide state (GTP/GDP) on polymerization.

The remainder of the paper is organized as follows. In Section 2, we discuss the self-organization process in MTs, as well as their dynamic instability and their interactions with MAPs. In Section 3, the principles of swarm engineering are presented, followed by the agent design and interaction rules. Section 4 introduces the simulator design, configuration space and simulation parameters, while the simulation results and analytics obtained from the interaction of GTPs are presented in Section 5. Section 6 provides a discussion of results and conclusions.

## 2. Self-Organization in Microtubules

Microtubules are complex networked protein structures responsible for many crucial intracellular processes in eukaryotic cells. These are highly dynamic polymers undergoing continuous assembly and disassembly. Structurally, MT’s are approximately 25 nm in diameter and have varying lengths [20]. They resemble hollow cylinders with dimers arranged on its walls.

**Figure 1:**
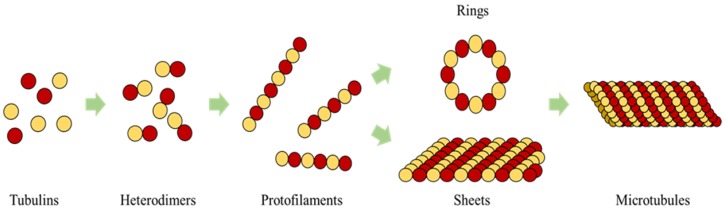
Self-organization of Tubulins, Heterodimers, Protofilaments, Sheets, Rings, intermittent structures leading to Microtubule formation.

The building blocks of the tubules are heterodimers and each heterodimer has a pair of α and β tubulins. These dimers attach in the cytoplasm to form long strings called protofilaments, with alternating α, β monomers [1] and these substructures are understood to undergo polymerization forming strings, rings and sheets as intermittent structures leading to the formation of MTs [21] as shown in Figure 1.

### 2.1. Microtubule Synthesis and Dynamic Instability

Synthesis of MTs starts from Microtubule Organizing Centers (MTOC) and in most animal cells Centrosomes act as MTOCs [22]. However, depending on the cell type, nucleation can happen in other sites [23]. Bartolini et al. [24] proposed the formation of non-centrosomal MT arrays as a three-step process in some animal cells and fungi. These steps involve creation, transport and arrangement of tubulins. Generally, these tubulins are released in the centrosomes or other sites like basal body [25] or from the breakage of other MTs itself.

Centrosomes are made of Centrioles with cartwheel structures, where a pair of them are arranged perpendicular to each other. They also hold the negative ends of the MTs and the growth of the MTs is from the opposite ends through the association of GTP bound dimers suspended in the cytoplasm and a tubulin in the Centrioles participates in nucleation and anchoring MTs [26].

GTPs bond with dimers activating them for polymerization and it loses a water molecule (hydrolysis), thereby changing to a Guanosine-diphosphate (GDP). This induces stresses in bonds [27] leading to a bond breakage, and hence the growth or shrinkage of an MT is dependent on the ratio of the rate of polymerization to the rate of hydrolysis. A ratio > 1 favors growth, which is dependent on the GTP cap at the growth end, as active dimers initiate polymerization [1] and thus by controlling the GTP cap, the growth and shrinkage of MTs can be controlled.

**Figure 2:**
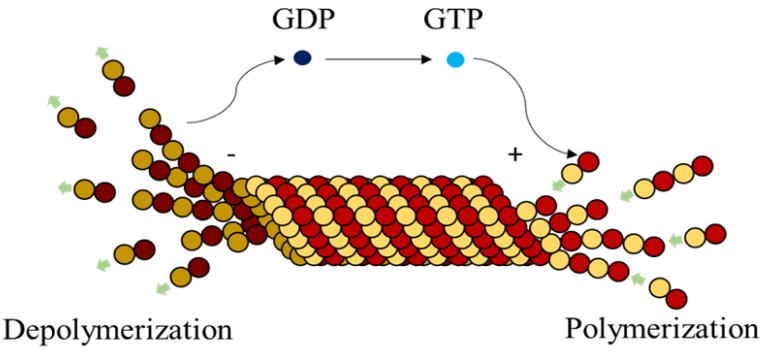
Active dimers associated with Guanosine-triphosphate (GTPs) polymerize to form microtubules (MTs). Simultaneously, GTPs associated with dimers hydrolyze to Guanosine-diphosphate (GDPs) leading to depolymerization (treadmilling).

Further, MTs display unique property of dynamic instability [1,2,28] where they undergo continuous assembly on the positive ends and disassembly on the negative ends simultaneously. Desai et al. [1] discussed MT polymerization [1] and also conceptualized the process of dynamic instability, where the MT dynamically alters between polymerization and de-polymerization influenced by catastrophe and rescue factors. Dynamic instability, on the other hand, is also indirectly controlled by the hydrolysis of GTP forming a GDP, inducing stresses in the bonds. This conversion weakens the bonds between the dimers and hence disassembly [29] takes place from the negative end, as illustrated in Figure 2. A single MT can sometimes alternate between cycles of assembly and disassembly depending on the MAPs it is associated with, a process also called treadmilling [28].

### 2.2. Microtubule Associated Proteins (MAP)

Microtubules are known to demonstrate complex organizations and intracellular functions by associating themselves with various MAPs. Some of these include chromosome separation during mitosis [3], organelle transport [30] and cell locomotion through cilia and flagella [1,31].

MTs’ dynamic instability controlled by MAPs was investigated by Drewes et al. [31]. In their work, they also investigated another set of proteins called MTaffinity-regulating kinases (MARKs) that are responsible for the detachment of MTs’ increasing instability through phosphorylation. These researchers have discovered many MAPs since and each discovery led to a new hypothesis on their functions. Some of these MAPs functions have been involved in guiding MTs’ growth towards selective targets like chromosomes in the cytoplasm during mitosis [32], in bundling multiple MTs or maintaining gaps between the fibers [33] and in varying the strength of bonds between dimers. For example, the MAP, CLASP [34,35] de-polymerizes MTs at a more rapid rate than normal, while a STOP holds the tubulins [10,36] from depolymerizing faster. Kinesin and dynein motor proteins [30] walk towards positive and negative polarities respectively during organelle transport and an XMAP215 increases the rate of polymerization [31,37]. Tom Hays et al. [30] investigated intracellular organelle transport by kinesins in neurons and found that these proteins are guided by the polarity gradient in MTs for their directional walking while moving the organelles.

## 3. Approach and Framework

Self-organization is an orderly process spontaneously arising in a system from local interactions of the parts of a disordered system. It is a decentralized process and hence no central agent controls it [38,39,40]. This phenomenon is observed in many physical, chemical and natural processes like swarming. In a review on swarm robotics by Brambilla et al. [14], self-assembly was classified under the subclass of spatially organized behaviors in collective behaviors alongside aggregation, pattern formation, object clustering and assembling. Spatially organized behavior is the distribution, organization and collective movement of robots in space in forming chains, patterns, aggregations and complex structures. Swarm engineering, on the other hand, is the application of knowledge, science and engineering design principles in modeling and developing swarm intelligent systems [41]. This area still being in its infancy, has seen significant developments in recent times through its applications in the field of robotics.

The concepts of Swarm Engineering (SE) in combination with self-organization principles are adopted in this study. Rules related to the geometry, environment and interactions between the tubulins and MAPs (agents) were developed according to the guidelines provided for the design of swarms [14,42] in combination with self-organization principles [40]. The swarm agents were designed to be autonomous, with an ability to sense and interact with the environment without any centralized control, while also maintaining coordination for task accomplishment [14,42,43]. At the agent level, we combine the general rules of self-assembly with SE, where the geometry, interactions and environment parameters at each level are precisely defined [39,40].

In the current study, we focus on demonstrating the formation of heterodimers, rings and protofilaments starting from α and β tubulins. Agent-based modeling was adopted and the agents were segregated into different levels based on their geometry and interactions. We modeled our agents in a behavior-based design approach [14], which focuses on understanding the behavior and processes occurring at the tubulin level. This is a trial and error process, in which the behavior of the agents was altered until the desired global configuration was attained. Also, our models were designed at the microscopic level, in which the agents are individually analyzed. This contrasts with a macroscopic model, where the whole system is analyzed through a set of differential and rate equations [14].

The rules designed are for forming protofilaments and ring sub-structures in a constrained 3D/2D space. We extended the current framework by adding a bond-breaking rule and thus introducing stochasticity into the system, caused by GTP to GDP conversions. The novelty of our research contribution is the development of a simulator for testing the proposed model. This simulator was developed in a Unity game engine, which provided an ideal environment for real-time simulations. Performance data for rings and protofilaments was logged for quantitative analysis.

### 3.1. Tubulins as Agents

In the current framework, we segregated all the proteins (tubulins and MAPs) into three types based on their functions as primary, secondary or control and functional agents, based on their levels of assembly. Primary agents at any level are the basic building block agents and any assembled primary agent at a lower level becomes a primary agent at a higher level. To demonstrate this, let us consider the general hypothesis regarding MT formation. At the basic level, both α and β tubulins act as primary agents. After the formation of heterodimers from tubulins, the definition is moved to the next level agents where the heterodimers are treated as primary agents. Similarly, this progresses through protofilaments, sheets and tubules, where each of these act as primary agents in that level.

Secondary or control agents influence the behavior of primary agents directly or indirectly. Each control agent is associated with unique functionality and can also change its state based on its association with other agents. For example, GTPs and MAPs acting on dimer level primary agents, vary bonding strengths, by which they demonstrate the dynamic instability behavior. These agents have specific effects on the primary agents and a group of different MAPs are responsible for achieving a target, say chromosome separation with kinetochore configuration [1].

**Figure 3:**
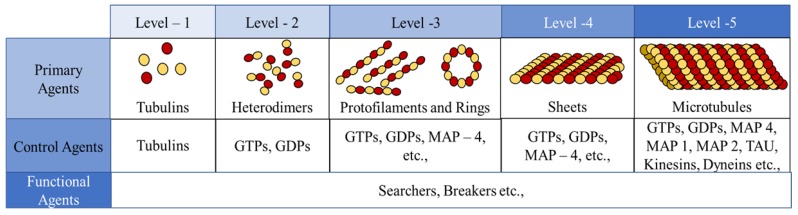
Segregation of agents into different types and levels in our proposed framework.

The third type are the functional agents which can work on the primary agents irrespective of their level, organization and association with other agents. Examples of these include searchers and breakers [44,45]. In the current study, we investigate the formation of protofilament and ring sub-structures through our framework. A summary of agent segregation in our current framework is presented in Figure 3. Currently, level 4 and 5 agents are not considered in this study.

### 3.2. Geometry

**Figure 4:**
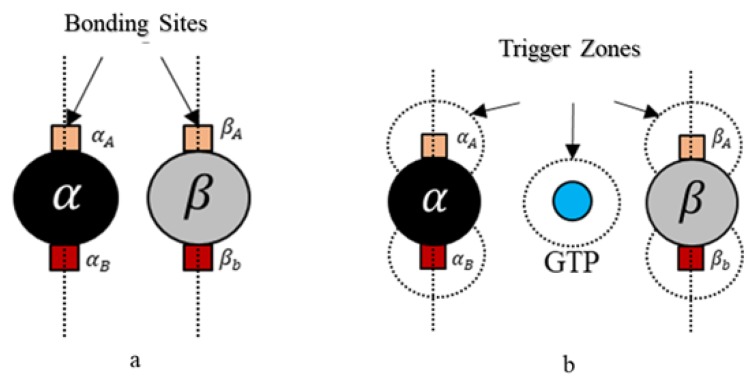
(**a**) α and β agents have two bonding site each and are labeled A and B sites respectively; (**b**) Region of activation also called trigger zone, where the bonding process is initiated.

Geometry, environment and interactions are the necessary parameters to be defined for having the desired self-organization behavior [39]. A spherical geometry was chosen, in order to avoid artifacts arising due to irregular geometries during simulations and also considering that globular proteins are generally spherical in shape in general. Moreover, we added additional tiny cube-like structures on the sphere surfaces for creating bonding sites as shown in Figure 4 and these locations are where one agent attaches itself with another.

Each tubulin has two bonding sites and a primary axis passing through the bond and sphere centers as shown in Figure 4. This axis is chosen as a reference for bond angles or azimuth angles, with respect to which variations in bond positions are made depending on the kind of interactions the tubulins has with different control agents. Initially, these bond angles are set to zero. A summary of agent properties is presented in Table 1.

### 3.3. Interaction Rules

Having defined the geometric parameters of the agents, we next define the interactions between different agents and the environment. In this section, we explicitly define these interactions for motion and interactions as rules and categorize them into preliminary and extended rules for the ease of simulations and analysis.

#### 3.3.1. Preliminary Rules

**Rule** **1** **-** **Environment.** *Tubulins and GTPs move randomly in a 2D space with a random force acting at regular intervals given by*

(1) $$F=\left\{\begin{array}{cc} 0 & if\;\Delta t\neq T\\ random(-F_{max},F_{max}) & if\;\Delta t=T \end{array}\right.$$

*where, F is the force acting on the tubulins and GTP agents, $F_{max}$ is the maximum allowed force magnitude. Δt is the time elapsed since last force application and T is the maximum interval time.*

Rule 1 defines the interaction of the tubulin agents with the environment [39]. Additional rules can be added to the current frame-work for simulating complex environments, like for example the motion of the particles governed by pH or heat gradients. These rules involve additional parameters and hence to make it less complex we maintain a constant environment (Rule 1) throughout the simulations and chose 0.1 s for ΔT to simulate Brownian motion patterns.

**Rule** **2.** *A bond is created between the agents if the following bond flag is true*

(2) $$Bond_{primary}=\left\{\begin{array}{cc} true & \mathrm{if}\;\mathrm{one}\;\mathrm{of}\;\mathrm{the}\;\mathrm{agents}\;\mathrm{is}\;\alpha \;\mathrm{and}\;\mathrm{the}\;\mathrm{other}\;\mathrm{is}\;a\;\beta \;-\mathrm{tubulin}\\ & \mathrm{are}\;\mathrm{free}\;\mathrm{to}\;\mathrm{bond}\\ false & \mathrm{otherwise} \end{array}\right.$$

Here, we define bonding sites as A and B explicitly in α and β–tubulins, as shown in Figure 4. Further, an ‘A’ site in α, can only bind with a ‘B’ site in β-tubulin and vice versa. The bonds are created only when they are close within the triggering regions as shown in Figure 4. Rule 2 was designed based on the formation of heterodimers from tubulins as explained in Section 2. It must be noted that these heterodimers are inactive and cannot bond with other dimers unless they are activated by GTPs as defined in Rules 3a and 3b.

We define rule 3, in two different ways based on the type of final shape desired. Each of these rules is based on the change of bonding locations due to interactions with GTPs as proposed by Ravelli et al. [46] and Akhmanova et al. [27]. This idea of relocating the bonds on the spheres draws inspiration from the work of Teich-McGoldrick et al. [47], in which they propose a model through bonding patches on spheres, which self-organize to form honeycomb structures. The details of these rules are presented in two parts.

**Rule** **3a** **(Protofilaments).** *An inactive dimer on bonding with a GTP is activated as per the following rule*

(3) $$Activation_{dimer}=\left\{\begin{array}{cc} true & \mathrm{if}\;\mathrm{the}\;\mathrm{current}\;\mathrm{dimer}\;\mathrm{is}\;\mathrm{inactive}\\ & \mathrm{and}\;P_{random}\leq P_{GTP}\\ false & \mathrm{otherwise} \end{array}\right.$$

**Figure 5:**
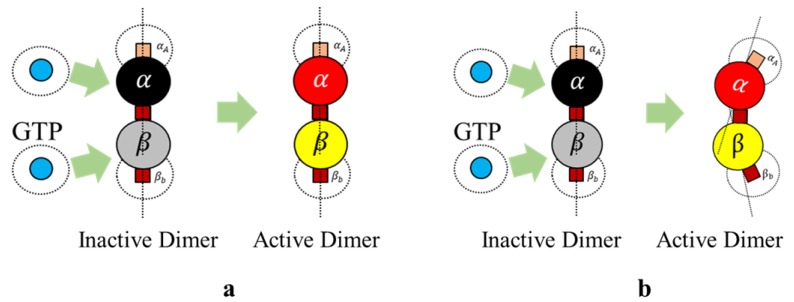
(**a**) Activation of dimer by GTP in protofilaments according to Rule 3a; (**b**) Activation and displacement of bonds in a dimer according to Rule 3b.

In rules 3a and 3b, it is required that two GTPs activate each of the tubulins (α and β). However, we associate this bonding with a probability function, where, $P_{GTP}$ is a preset value and $P_{random}$ is a random value between 0 and 1 and the tubulins are activated upon meeting these conditions. In our simulations presented later, we change the color of tubulin according to Table 1. An illustration of GTP interactions is presented in Figure 5.

**Figure 6:**
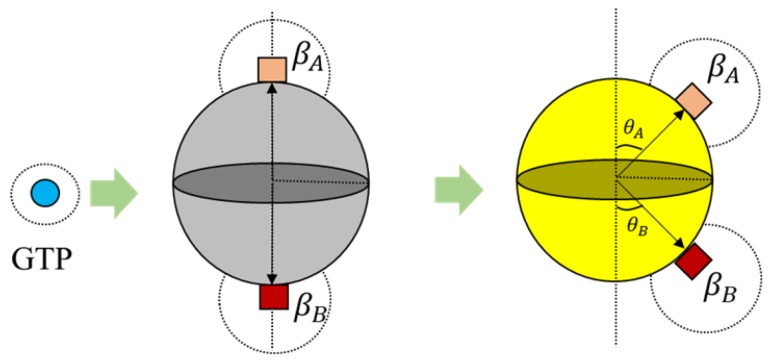
Bond displacement during activation of dimers as per Rule 3b for ring formation.

Further, the bonds in 3D placed on a spherical surface can be rotated with respect to their azimuth and elevation angles as shown in Figure 6 for rings. But, for our current simulations, the elevation angle was not considered as the motion of the agents is limited only to two dimensions. Now, these angles are computed with respect to the axis as shown in Figure 6.

The condition in Equation (3) is applicable for the ring formation as well. However, the bond angles $\theta_{A}$ and $\theta_{B}$ change, as shown in Figure 6. These values were chosen prior to running simulations, based on the ring size and the number of members required. For the current simulations, this value was set at 30°, accommodating 12 members in the ring. An illustration of transformation in bond angles in dimers is shown in Figure 5.

**Rule** **3b** **(Rings).** *An inactive dimer on bonding with a GTP is activated as per Rule 3a and the bonds are displaced.*

**Rule** **4.** *An active dimer bonds with another active dimer, when the following condition is met*

(4) $$Dimer_{bond}=\left\{\begin{array}{cc} true & \mathrm{if}\;\mathrm{both}\;\mathrm{tubulins}\;\mathrm{in}\;\mathrm{the}\;\mathrm{current}\;\mathrm{dimer}\;\mathrm{are}\;\mathrm{active}\\ & \mathrm{the}\;\mathrm{other}\;\mathrm{dimer}\;\mathrm{has}\;a\;\mathrm{vacant}\;\mathrm{bonding}\;\mathrm{site}\\ & \mathrm{the}\;\mathrm{vacant}\;\mathrm{bonding}\;\mathrm{site}\;\mathrm{is}\;\mathrm{of}\;a\;\mathrm{complementary}\;\mathrm{tubulin}\\ false & \mathrm{otherwise} \end{array}\right.$$

**Figure 7:**
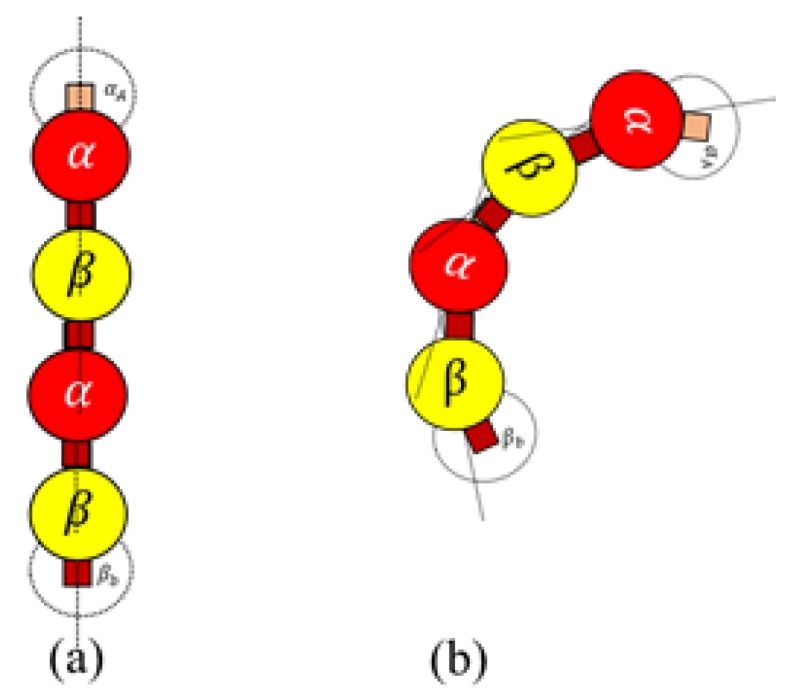
(**a**) Bonding between active dimers for protofilament formation (Rule 3a and Rule 4); (**b**) Bonding between active dimers for ring formation (Rule 3b and Rule 4).

When $Dimer_{bond}$ is true, a bond is created between two heterodimers as shown in Figure 7. This rule is for polymerizing dimers assembled in Rules 3a and 3b, for the formation of protofilaments and rings.

#### 3.3.2. Extended Rules

Apart from the regular rules for assembly discussed in the previous section, we also propose two additional rules in line with the bond breakage in protofilaments and hydrolysis of GTP to GDP and GDP to GTP. These rules are currently only for the protofilament formation and breakage; however, they can also be extended to the formation of rings.

**Extended** **Rule** **1.** *A GDP turns into a GTP according to the following rule,*

(5) $$Activation_{GTP}=\left\{\begin{array}{cc} true & \mathrm{if}\;\mathrm{the}\;\mathrm{current}\;\mathrm{state}\;\mathrm{is}\;\mathrm{GDP}\\ & \mathrm{and}\;\mathrm{if}\;Duration_{GDP}\geq Limit_{GDP}\\ false & \mathrm{otherwise} \end{array}\right.$$

A GTP is formed from a GDP by associating with a water molecule. To reduce the complexity of introducing water molecules, we introduced a duration function where a GDP converts to a GTP after a certain duration $Limit_{GDP}$. A duration time, $Duration_{GDP}$ is maintained for a GDP since its spawn and upon crossing the $Limit_{GDP}$, the state changes to a GTP.

**Extended** **Rule** **2.** *A terminal dimer breaks from a protofilament according to the following condition*

(6) $$Dimer_{Break}=\left\{\begin{array}{cc} true & \mathrm{if}\;\mathrm{the}\;\mathrm{current}\;\mathrm{dimer}\;\mathrm{is}\;\mathrm{bonded}\;\mathrm{to}\;\mathrm{another}\;\mathrm{dimer}\\ & \mathrm{and}\;\mathrm{if}\;P_{random}\leq P_{computed}\\ & \mathrm{and}\;\mathrm{if}\;\mathrm{the}\;\mathrm{dimer}\;\mathrm{is}\;a\;\mathrm{terminal}\;\mathrm{one}\\ false & \mathrm{otherwise} \end{array}\right.$$

The value, $P_{computed}$ is computed as follows

(7) $$P_{computed}=1-{\left(1-P_{break}\right)}^{Proto_{length}}$$

**Figure 8:**
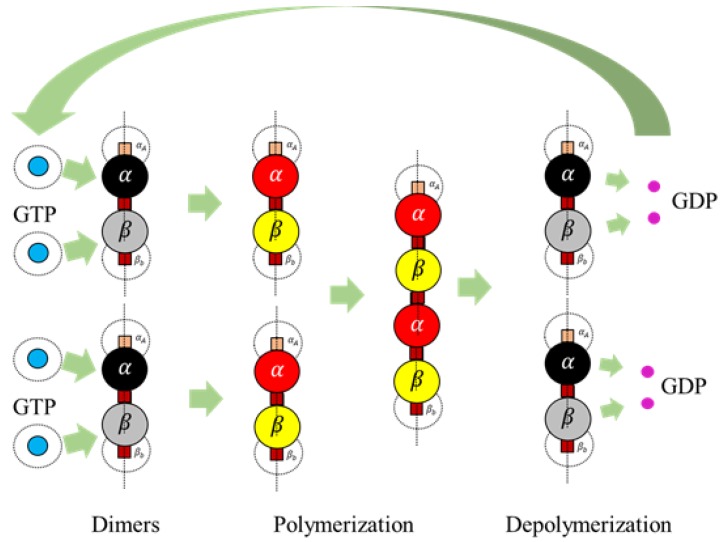
The complete cycle from activation of dimers to polymerization and depolymerization applying extended rules.

In Equation (7), $P_{break}$ is a constant, given as user input and $Proto_{length}$ is the length of the polymer, the dimer is present in. From the equation, the probability of breakage increases with the increase in length and hence longer lengths tend to break dimers at the terminal ends, with a higher probability. After a bond breakage, GDPs are released by the dimers mimicking the hydrolysis process and turn inactive getting ready to associate themselves with GTPs. GDPs turn into GTPs as per the extended rule 1. An illustration of the complete process is presented in Figure 8.

**Figure 9:**
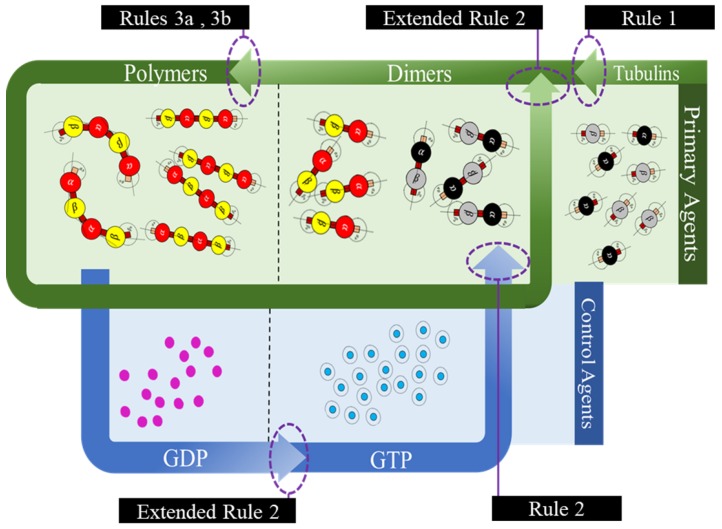
A summary of agent segregation and interaction rules.

We summarized all the rules in the form of a flow diagram in Figure 9 and we implemented them on a simulator discussed in the next section.

## 4. Simulations and Evaluation

### 4.1. Simulation Environment

We simulated our proposed framework in a Unity game engine [16], widely used for game development and simulations to mimic the self-organization of substructures of MTs. It has a strong physics engine and can execute programs in a parallel real-time environment. This was our best choice as it supports our multi-agent strategy by allowing a single code to run on all the game agents, instead of time-based allotment of a processor for each agent, as done in other sequential ways. Another advantage of running swarms in unity is scalability, where the number of swarm agents can be increased to large numbers without compromising real-time performance.

### 4.2. Configuration Space

**Figure 10:**
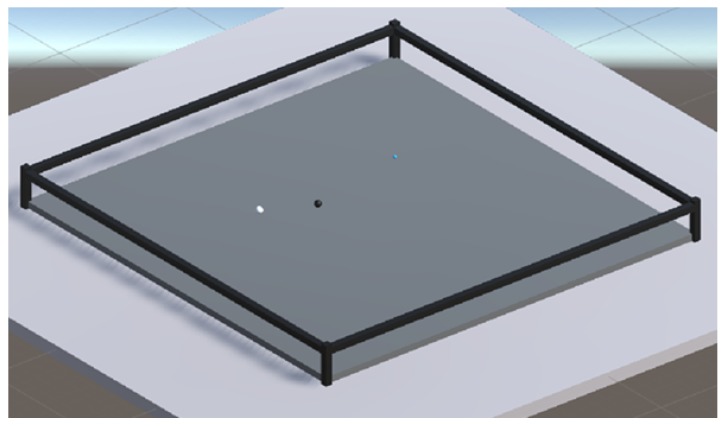
Sample Configuration space, with α (gray),β (black)–tubulins and GTP (teal) agents.

We designed a 3D configuration space in Unity but limited most of the movements of the swarm agents on to a 2D plane. The configuration space is a transparent tray, designed to constrain the motion of agents along the vertical axis using colliders. Also, in the design, we ensured that the agents do not fly away during simulations by adding invisible colliders on the walls. The dimensions of the tray were maintained at 3 × 3 units and all the agents were initialized at random locations in each iteration. A sample configuration space and agents is shown in Figure 10.

### 4.3. Simulation Parameters

The first two sets of simulations were run by varying GTP population and GTP bonding probabilities for the formation of protofilaments according to Rule 3a and with no bond break rules (extended rules). GTP bonding probability is a factor assigned to GTPs deciding their bonding ability with an inactive dimer upon interaction. This study was conducted to understand the factors affecting the polymerization of protofilaments.

Our first set of simulations were on a population (molecules) of 300 each of α and β tubulins. The GTP populations were varied between 200 and 1200, for a constant GTP bonding probability of 1. In the second simulation, we kept the GTP constant at 600 equating the total count of α and β–tubulins and varied the bonding probability on from 0.05 to 1. A summary of these experiments is presented in Table 2.

The selection of population values are based on computational complexity and simulation times. Fewer agents took longer times for self-assembly of substructures. However, with larger number of agents the simulations took longer time. Also, the selection of population ratios of GTPs was based on the possible scenarios arising in the cell environment. Hence, we arrived at these values after a systematic study of α and β population variation between 100 to 500 agents/molecules. However, for the current simulations a population of 300 for each of α and β was considered.

The third set of simulations were run on the formation of rings (Rule 3b) for 300-α, 300-β tubulins and 600–GTPs. This was followed by a final set of simulations with the extended rules incorporated along with Rule 3a for the formation of protofilaments. In these simulations, we varied the $P_{break}$ between 0 and 0.5 in 0.1 intervals.

We conducted three trials for each simulation parameter combination. Our simulator was designed to record the inactive dimer count, active dimer count, unbonded GTP, GDP count, the maximum length of protofilaments and number of protofilament chains within the configuration space for each iteration. In all our simulations, frame count was considered as an iteration and a fixed frame update of 0.01 s was set. Simulations 1, 2 and 3 were run for a total of 120,000 iterations and simulation 4 was run for 100,000 iterations.

We ran all the simulations on a workstation with an i7 (6700) processor, clock speed set at 3.70 Ghz, 32 GB RAM, NVIDIA 1080 GTX Founder’s edition GPU with a total of 2560 Cuda Cores. We logged all the analytics data into a text file which was later processed in MATLAB 2019a.

## 5. Results and Discussion

**Figure 11:**
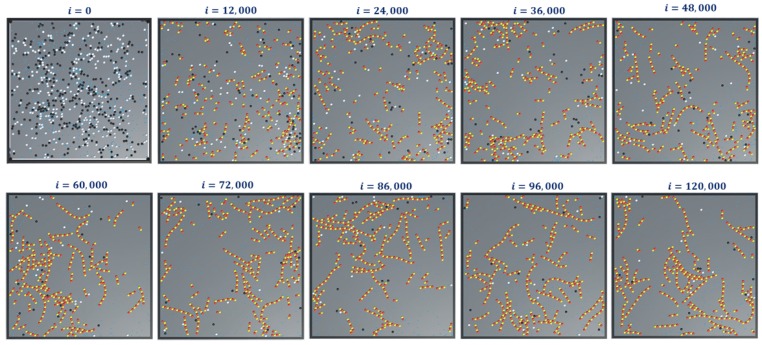
Snapshots of simulation—2 showing formation of protofilaments from tubulins. The simulation was run for 300 each of α and β tubulins, 600 GTPs, $P_{GTP}=1$ and 120,000 iterations and with no extended rules. Long chain protofilaments can be observed in the end.

**Figure 12:**
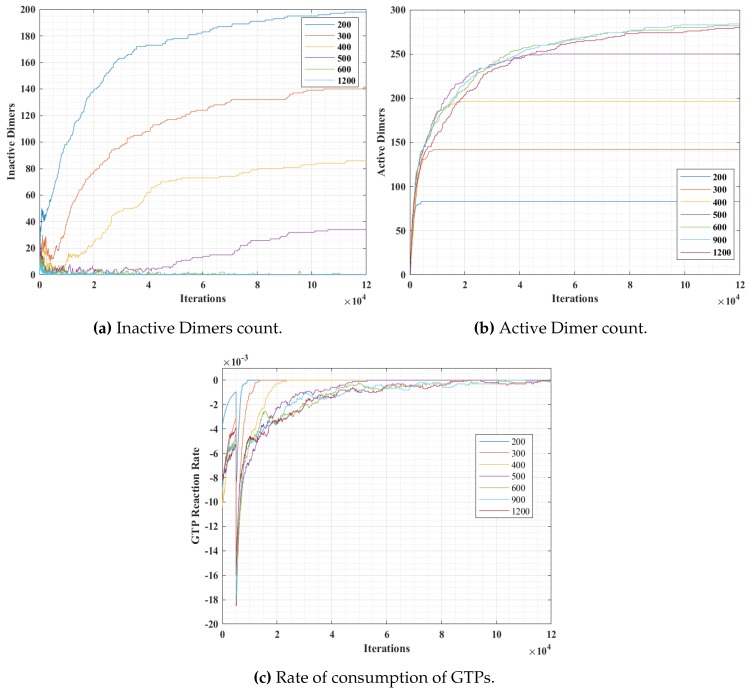
Analysis results of simulation 1, with varying GTP population over iterations.

**Figure 13:**
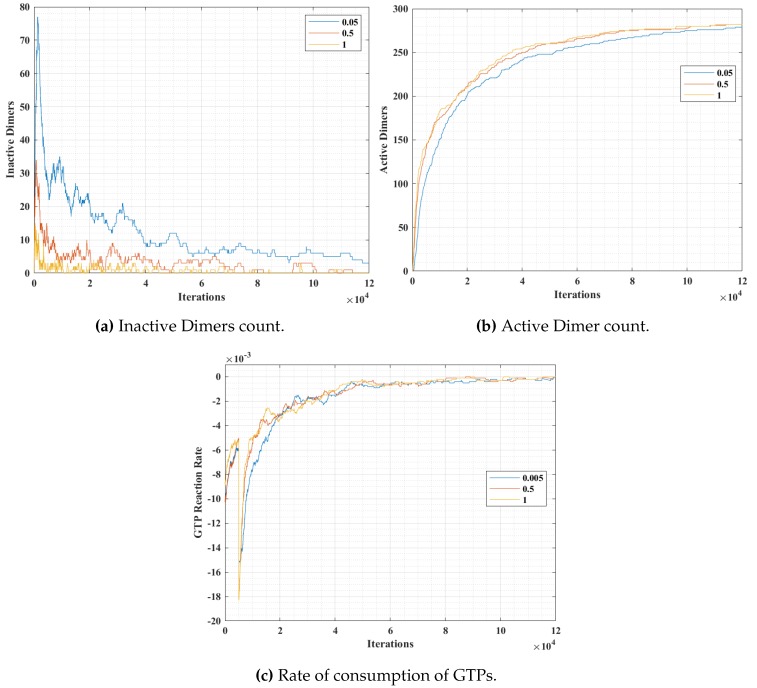
Analysis results of simulation 2, with varying *P_GTP_*.

The simulation snapshots during protofilament (Rule 3a) formation, for 300- α, 300-β, 600 GTPs, $P_{GTP}=1$ and with no breaking rules (or $P_{break}=0$) is presented in Figure 11. With the same settings, the GTP population and bonding probabilities were varied according to the values presented in Table 2 and as explained in Section 4.3. The graphs for inactive dimer count, active dimer count and the rate of consumption of GTPs for varying population and probabilities are presented in Figure 12 and Figure 13 respectively. The rate consumption of GTPs was computed by taking a successive difference on a moving average (1000 point window) data of GTP count. Also, a higher magnitude (lower value) denotes a higher rate.

In these set of simulations, we varied the population of GTPs with a constant population of α and β tubulins. From the graphs presented in Figure 12, we can observe that the number of inactive dimers grew at a faster rate for a lower GTP count than the total population of the tubulins. However, the population count has no significant effect on inactive dimer count for the GTP population greater than the total population of tubulins as compared to a lesser population. A slight dip in the inactive dimer count can be observed initially, which also corresponds to the rate of consumption of GTPs as shown in Figure 12c.

This can be attributed to the rapid formation of inactive dimers in the beginning from tubulins and hence a rapid consumption of free GTPs. It can be noted that in Figure 11b, the active dimer count reaches a saturation, for populations of GTPs lower than the total tubulin count, as it is obvious that there are no further GTPs available. Also, no significant change in active dimer count was observed for populations of GTPs, higher than the total GTP count. This also corresponds with the GTP rate consumption graph, where the rate consumption of GTPs rapidly settles to zero, for populations less than 0 and took time for the rest.

In simulation 2, we varied the GTP bonding probability ($P_{GTP}$) with a constant population of α and β tubulins and GTPs at 300, 300 and 600 units respectively. The results are presented in Figure 13. The variation of probability was for three different values at 0.05, 0.5 and 1, where a probability of 1 corresponds to an immediate bonding upon interaction and a probability of 0.05 corresponds to a rare bonding possibility on interaction with an inactive dimer tubulin.

From Figure 13a, it can be observed that the inactive dimer count dropped rapidly, in the case of a higher $P_{GTP}$ value. However, the final values of inactive dimers settled down eventually for lower probabilities. This is also complemented by the active dimer count graph in Figure 13b, where the count after a large number of iterations settled, irrespective of the $P_{GTP}$ value. A similar trend in the rate of consumption to that of in Figure 12c can be observed in Figure 12c. However, the value peaks for a relatively higher $P_{GTP}$ value.

The results from simulations 1 and 2, indicate that the population density of GTP significantly affects the rate of protofilament formation. However, the effect is diminished beyond a certain count. Also, it is observed that at a higher GTP concentration, protofilament formation rate is also determined by the rate of inactive dimer formation from the tubules. This validates the requirement of maintaining GTP concentration above a critical value for sustained polymerization in MTs [48]. This polymerization can also be accelerated further by the addition of agents mimicking MAPs like XMAP215 [31,37].

**Figure 14:**
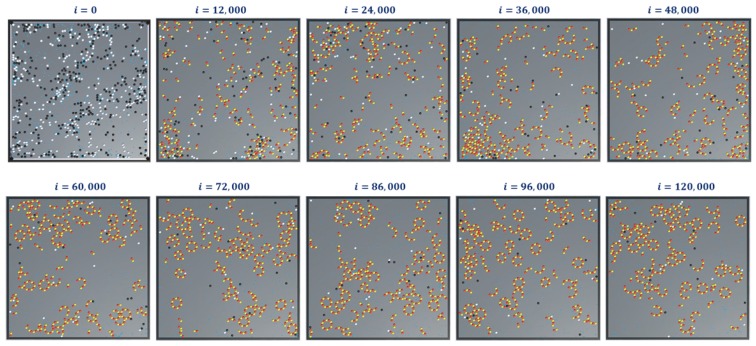
Snapshots of simulation-3 showing formation of rings from tubulins. The simulation was run for 300 of each of α and β tubulins, 600 GTPs, $P_{GTP}=1$, 120,000 iterations and with no extended rules. Multiple closed and open rings can be observed in the end.

Finally, we present snapshots of simulation-3 for ring formations in Figure 14. This was for 300- α and β tubulins, 600 GTPs with $P_{GTP}=1$ and with no breaking rules (or $P_{break}=0$). For this set of simulations, the analysis graphs for the formations are found to be similar to that of the formation of protofilaments shown in Figure 12 andFigure 13 and hence are omitted.

**Figure 15:**
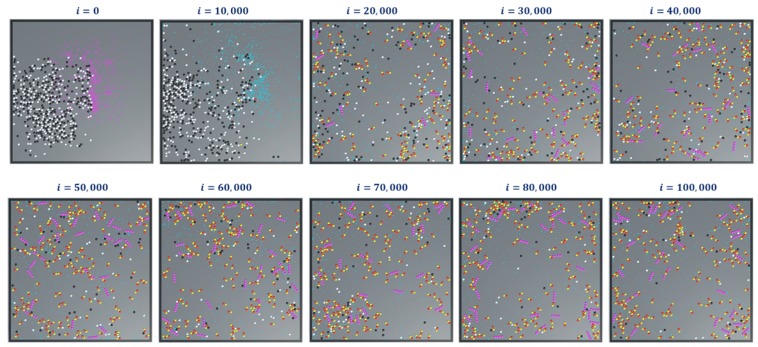
Snapshots of simulation-4 showing formation of protofilaments from tubulins, with extended rules. The simulation was run for 300 of each of α and β tubulins, 600 GTPs, $P_{GTP}=1$ and 100,000 iterations.

In simulation 4, we added the extended rules to protofilament formation simulations in 1 and 2. The simulation snapshots are shown in Figure 15. At the beginning of the simulation (i = 0), we started with GDPs instead of GTPs with $\Delta T_{GDP->GTP}=3s$ (Ref. Table 2). This means that a GDP turns into a GTP automatically after 300 iterations $\left(\frac{3s}{0.01s/iteration}=300\mathrm{iterations}\right)$. This can be observed in the consecutive iterations where the GTPs are represented in teal color. Also, the terminal dimers in a protofilament chain are represented in purple. After 100,000 iterations, very few protofilaments of length greater than four dimers are observed compared to the long chains in simulations 1 (Figure 11) and 2 (Figure 14).

**Figure 16:**
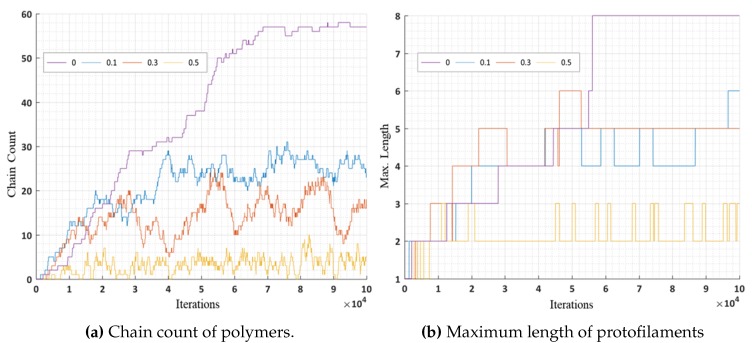
Analysis results of simulation 4, with constant *P_GTP_* = 1 and a varying *P_break_* over iterations.

In these simulations, we further analyzed the number of chain counts and longest chains formed for varying bond breakage probabilities are presented in Figure 16a,b respectively. A lower probability of breakage formed longer chains and maximum chain count, whereas a higher bond breakage probability formed smaller chains with lesser chain count. We can relate these variations in probabilities to the presence of MAPs (CLASP) [34,35] controlling the rapid bond breakage.

## 6. Conclusions and Future Work

In this study, we presented a framework for simulating substructures self-organization in MTs through the principles of swarm engineering. In this framework, we segregated the α and β–tubulins and GTP molecules into different levels and groups based on the type of interaction and the complexity of the base structure. We proposed an agent-based modeling approach, through which α and β tubulins were modeled to form dimers, protofilaments and ring substructures. We designed a 2D simulator and our simulations demonstrated the formation of protofilaments and rings. These sub-structures resemble geometries of the real protofilaments and rings suspended in the cytoplasm of a cell [1,49,50].

Our analysis of simulation data showed that GTP population density played a crucial role in accelerating the polymerization process. However, the effect is insignificant after a specific population size and this indirectly validates the participation of other MAPs in accelerating polymerization. In a different scenario, we assimilated accelerated de-polymerization forming shorter protofilament chains through extended rules, also indirectly validating the effect due to MAPs [9,10].

The current simulator was developed in a 3D environment and the movement of the molecules was restricted to a 2D space for reducing the simulation complexity as we analyzed only the formation of Level 2–3 sub-structures. Considering the recommendation made by Jamie et al. [19] about the effect of spatial dimensionality, we plan to extend the framework in 3D (Level 4) in the future to form complete MTs from the current substructures (Level 1-3). We also plan to include the effect of MTOCs, Golgi apparatus by adding new environments and interaction rules. Our current framework is scalable and has the capability of handling thousands of agents. We plan to take advantage of this aspect to study the other complex processes like dynamic instability, the effect of MAPs on MT stability and organelle transport in the future. Also, the current system is expected to demonstrate similar outcomes presented in the current work, while extending it to higher level structures.

## Figures and Tables

**Table 1:** Summary of Agent properties.

	Tag	Alpha	Beta	GTP
**Geometry**	Shape	Sphere	Sphere	Sphere
	Bonding Sites	2	2	0
	Color	Black (Red)	Gray (Yellow)	Cyan
	Initial Bond Angles	(0,0)	(0,0)	-
**Motion**	DOF	2	2	2
	Forces	Random	Random	Random
	Boundaries	Yes	Yes	Yes
	Collision	Yes	Yes	Yes
**Interacting Agents**	Primary Agents	- Tubulin	- Tubulin	-Tubulin
	MAPs	GTP	GTP	-

**Table 2:** Simulation and Parameter Combination Summary.

	Parameter	Value
**Configuration Space**	Dimensions	3 × 3 units
**Simulation–1**	Shape	Protofilaments
	Tubulins	300-α, 300-β
	$P_{GTP}$	1
	GTP Population	200, 300, 400, 500, 600, 900, 1200
**Simulation–2**	Shape	Protofilaments
	Tubulins	300-α, 300-β
	$P_{GTP}$	0.05,0.5,1
	GTP Population	600
**Simulation–3**	Shape	Rings
	Tubulins	300-α, 300-β
	$P_{GTP}$	1
	GTP Population	600
**Simulation–4**	Shape	Protofilaments (with extended rules)
	Tubulins	300-α, 300-β
	$P_{GTP}$	1
	$\Delta T_{GDP->GTP}$	3s
	GTP Population	600
	$P_{break}$	0, 0.1, 0.2, 0.3, 0.5
